# Complete chloroplast genome sequence of *Bletilla striata* (Thunb.) Reichb. f., a Chinese folk medicinal plant

**DOI:** 10.1080/23802359.2020.1770138

**Published:** 2020-06-01

**Authors:** Ziping Cai, Hongxia Wang, Guoxiang Wang

**Affiliations:** a Gansu Academy of Agricultural Sciences, Institute of Chinese Herbal Medical, Lanzhou, Gansu, People’s Republic of China; b Engineering Laboratory of Germplasm Improvement and Quality Control of Gansu Province, Lanzhou, Gansu, People’s Republic of China

**Keywords:** Chloroplast genome, phylogenetic analysis, *Bletilla striata* (Thunb.) Reichb. f.

## Abstract

*Bletilla striata* (Thunb.) Reichb. f. is one of the commonly used traditional Chinese medicine with tuber as medicine. We report herein the complete chloroplast genome sequence of *Bletilla striata* (Thunb.) Reichb. f. It has a length of 159,491 bp, which contained a small single-copy (SSC) region of 18,778 bp and a large single-copy (LSC) region of 87,139 bp, separated by two copies of an inverted repeat (IR) of 26,787 bp. The chloroplast genome contains 114 unique genes, including 80 PCG, 30 tRNA, and 4 rRNA genes. In addition, 18 genes contained one or two introns, which of those including 10 PCG genes possess a single intron, and 2 PCG genes harbor two introns; and 6 tRNA genes harbor a single intron. In this study, *Bletilla stariata* is sister to *Bletilla formosana* and clustered within the group consisting of the species that belong to Orchiidaceae.

*Bletilla striata* (Thunb.) Reichb. f. is one of the commonly used traditional Chinese medicine with tuber as medicine (He et al. 2017; Xu et al. 2019), Especially, in the northwest region high-quality *B. striata* (Thunb.) Reichb. f. is grown, which is used by the pharmaceutical industry (Bai et al. 1993). The root tuber of *B. striata* (Thunb.) Reichb. f. has been used over the years in traditional medicine to treat myriad of diseases. Medicinal plants are widely used for the treatment of human diseases, there is a growing interest in *B. striata* (Thunb.) Reichb. f. But, with the increasing demand of *B. striata* (Thunb.) Reichb. f. in the market, the wild resources are gradually exhausted. *Bletilla striata* (Thunb.) Reichb. f. is of high economic value because of the utilization of wild herb medicine species with medicinal values in northwest China. The data of complete chloroplast genome will serve as a foundation for species identification, germplasm diversity, genetic engineering of *B. striata* (Thunb.) Reichb. f. In this study, we aim to establish and characterize the complete chloroplast (cp) genome of *B. striata* (Thunb.) Reichb. f., and provide additional effective data for the phylogenetic study of Orchiidaceae in the future.

In this study, fresh leaves of *B. striata* (Thunb.) Reichb. f. were collected from solar greenhouse of Gansu Academy of Agricultural Sciences of China, located at 103.6848E, 36.1001N. The voucher specimen (No. HM1912186) was deposited at the Engineering Laboratory of Germplasm Improvement and Quality Control of Gansu Province, Lanzhou, Gansu, China. The procedure of DNA isolation, genome sequencing and data processing all were followed according to the previous research by Yan et al. (2019).

The complete chloroplast genome was annotated with *B. formosana* (MN526744) and *B. ochracea* (KT695602) as reference and has been submitted to GenBank with the accession number of MT193723. The chloroplast genome of *B. stariata* is a circular quadripartite structure similar to major angiosperms chloroplast genomes (Hahn et al. 2013). It has a length of 159,491 bp, which contained a small single-copy (SSC) region of 18,778 bp and a large single-copy (LSC) region of 87,139 bp, separated by two copies of an inverted repeat (IR) of 26,787 bp.

The chloroplast genome contains 114 unique genes, including 80 PCG, 30 tRNA and 4 rRNA genes. In addition, 18 genes contained one or two introns, which of those including 10 PCG genes (atpF, ndhA, ndhB, petB, petD, rpl2, rpl16, rpoC1, rps12 and rps16) possess a single intron, and 2 PCG genes (clpP, ycf3) harbor two introns; and 6 tRNA genes (trnA-UGC, trnG-GCC, trnI-GAU, trnK-UUU, trnL-UAA and trnV-UAC) harbor a single intron.

The Bayesian phylogenetic tree was generated using TOPALi v2.5 (Milne et al. 2009) based on the complete chloroplast genome of *B. stariata* and 24 other species from Orchiidaceae and Epidendroideae. Further, the phylogenetic tree analysis showed that *B. stariata* was closely related to the genus *Bletilla formosana* (Figure 1).

**Figure 1.**
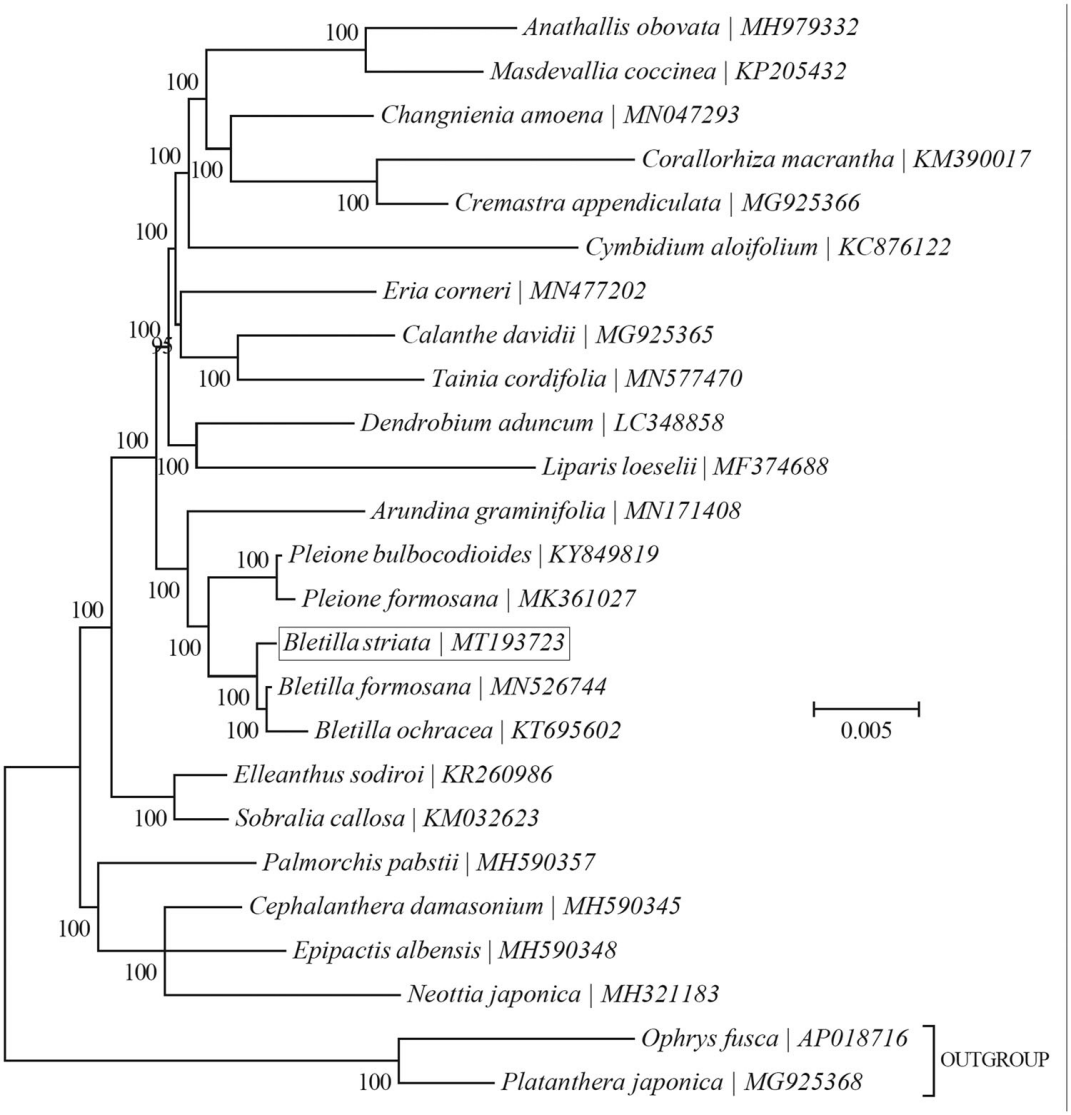
The phylogenetic tree (Bayesian inference) of *B. striata* (Thunb.) Reichb. f. and its related relatives based on the complete chloroplast genome sequences.

In this study, *B. stariata* is sister to *B. formosana* and clustered within the group consisting of the species that belong to Epidendroideae, and the determination of the complete plastid genome sequences provided a useful resource for new molecular data to illuminate the *B. stariata* evolution.

## Data Availability

We confirm that the data supporting the findings of this study are available within the article and its Supplementary material. The data that support the findings of this study have been deposited in the GenBank (https://www.ncbi.nlm.nih.gov/) with accession number MT193723.
